# Evaluating Chinese Mobile Health Apps for Ankylosing Spondylitis Management: Systematic App Search

**DOI:** 10.2196/27234

**Published:** 2021-07-14

**Authors:** Yuqing Song, Hong Chen

**Affiliations:** 1 West China School of Nursing/West China Hospital Sichuan University Chengdu China

**Keywords:** ankylosing spondylitis, app, eHealth, mHealth, smartphone, mobile phone

## Abstract

**Background:**

Patients with ankylosing spondylitis (AS), a chronic systematic inflammatory disease, require long-term treatment and management. Mobile health (mHealth) apps can deliver health services through mobile devices, facilitate long-term disease management, support patient–health care provider communication, and enable patients to engage in disease management. There are some apps targeted at patients with AS, but the feature and quality of these apps have not been systematically examined.

**Objective:**

The aim of this study was to identify existing, publicly available Chinese mHealth apps for AS management and to evaluate their features and quality.

**Methods:**

We systematically searched potential apps for AS management on the Apple and Huawei App Stores, using 4 search terms: *ankylosing spondylitis*, *spondyloarthritis*, *rheumatic disease*, and *arthritis*. Apps were included if they were in the Chinese language, targeted at patients with AS, could be downloaded and run on Android and/or iOS operating systems, and incorporated elements of disease management and/or patient education. We excluded apps that were not for patient use, not relevant to AS, or had not been updated since 2018. Apps that met the inclusion criteria were downloaded for final analysis. We formulated a list of app quality measures from and consistent with international guidelines for mHealth apps and AS management to evaluate the features and quality of the included app. The user version of the Mobile App Rating Scale (uMARS) was also used to rate the apps’ quality.

**Results:**

Of the 354 apps screened, 5 met the inclusion criteria and were included in our analysis. All apps were free, and most apps (4/5, 80%) had a privacy policy. Of the 5 apps, 1 (20%) involved medical professionals in the development process, 2 (40%) were developed by companies, and 2 (40%) were developed by medical institutions. All apps provided educational information about AS. Around half of the apps had functions like a basic information record (ie, users can input gender, age, disease history, etc) (n=3, 60%), patient–health care provider (and patient-patient) communication (n=2, 40%), symptom tracking (n=2, 40%), and information sharing (n=3, 60%). Only 1 (20%) app provided comprehensive functions that adhered to international guidelines for AS management and mHealth apps. The overall uMARS scores ranged from 2.7 to 4.2; only 1 app, with an overall uMARS score of 4.2, was considered as a high-quality app.

**Conclusions:**

Most apps lacked comprehensive functions for AS management. One high-quality app provided comprehensive functions to help patients manage their conditions. This study assessed and summarized the features and quality of the apps but did not evaluate their efficacy. Future studies should evaluate the feasibility and efficacy of these apps. International guidelines and regulations for the design, development, validation, and implementation of mHealth apps are needed in the future. Meanwhile, health care providers, patients with AS, and app developers should collaborate to develop high-quality, evidence-based apps that take into account patients’ needs and health care professionals’ perspectives.

## Introduction

Ankylosing spondylitis (AS) is a chronic systematic inflammatory disease that often causes structural impairment, functional disability, and impaired quality of life [1,2]. AS often affects young people at their most productive age (approximately 20-35 years) and has a lifelong impact on their life [1]. AS can interfere with work and schooling, and can impose substantial physical and social burdens on patients [3]. The guidelines of the European League Against Rheumatism (EULAR) recommend that AS requires long-term management, including pharmacological and nonpharmacological treatment, to control inflammation, prevent structural damage, and optimize function and quality of life [4]. The management of long-term conditions requires timely health care and treatment [5].

The traditional approach to AS management requires face-to-face rheumatology clinic appointments and treatment [6,7]. Patients with chronic diseases may have difficulty in attending regular rheumatology clinic appointments and obtaining timely treatment due to transportation difficulties, physical limitations, time constraints, and geographical barriers [8,9]. Moreover, China has a large population, with a broad geographical distribution of patients with AS; around 90% rheumatologists work at tertiary hospitals [10-12]. The barriers to receiving timely treatment and health care are much more prominent in China [11,13].

Mobile health (mHealth) has the potential to facilitate long-term disease management and deliver timely health care to hard-to-reach populations [14,15]. mHealth is defined as medical and public health practice supported by mobile devices, such as mobile phones, personal digital assistants, and other wireless devices [16,17]. mHealth apps can overcome barriers of time and geography to deliver health services through mobile devices and increase access to health care service [18]. Mobile apps offer a solution for patients with chronic diseases to monitor chronic conditions, support patient–health care provider communication, provide health advice, and enable patients to engage in disease management [10]. Many mHealth apps have been developed to help individuals with chronic diseases, such as asthma, rheumatoid arthritis, and inflammatory bowel disease, to manage their conditions [16,19-21]. Prior work has supported the usefulness of mHealth apps for enhancing disease management and improving clinical outcomes among patients with chronic diseases [15,22].

In China, 932 million people used smartphones to access the internet, and 3.59 million mobile apps were available as of 2020 [23]. Thus, mHealth interventions are increasingly accessible for Chinese people [10]. In the area of rheumatology, many studies revealed that mHealth apps for patients with rheumatoid arthritis have the potential to monitor their disease and support high-quality medical care [14,16,20,24]. Patients with AS may be one of the best target users of mHealth apps because AS affects young people (approximately 20-35 years) [10]. Young people are more likely to be acceptable to receiving an mHealth intervention [25,26].

Limited published works in the literature have focused on mHealth apps for AS management in China [7,27]. Although the Smart System of Disease Management (SSDM) was used for AS management, the characteristics and functions of the SSDM have not been clearly described in the literature [7,27]. Our search of app stores found that there are some publicly available apps for AS management despite limited published works. However, there is little information to users on which apps provide evidence-based tools [20]. A previous review revealed that many apps are not supported by research, do not provide evidence-based therapies, and do not follow clinical guidelines [28]. Kwan et al [29] reviewed English-language apps for monitoring disease activity in patients with spondyloarthritis and found only 2 high-quality apps. However, the characteristics and functionalities of Chinese mHealth apps for AS management have not been systematically evaluated (eg, in terms of star rating, privacy policy, evidence-based content, and functionality).

The purpose of this study was to identify existing, publicly available apps targeted at patients with AS for disease management, formulate a list of app assessment measures from and consistent with evidence-based guidelines to evaluate the features and quality of these apps, and rate app quality using the user version of the Mobile App Rating Scale (uMARS). This will help patients choose a high-quality app for managing their disease and identify gaps in the current apps available for AS management.

## Methods

### App Selection

We conducted a systematic search of all potential apps targeted at patients with AS in November 2020. Android and iOS are the two most popular smartphone operating systems among Chinese smartphone users [30]. The Huawei App Store is one of the biggest Android app stores in China [31]. Thus, we searched for iOS apps in the Apple App Store and Android apps in the Huawei App Store. Preliminary test searches were conducted to determine the search terms before the final search. To ensure all potential apps were screened, our final search terms included *ankylosing spondylitis* or *spondyloarthritis* or *rheumatic disease* or *arthritis* in both the Huawei and Apple app stores. The final search was conducted following the PRISMA (Preferred Reporting Items for Systematic Reviews and Meta-Analyses) guidelines for systematic reviews. Two reviewers independently downloaded potential apps to test devices for screening according to the inclusion and exclusion criteria. The inclusion criteria for the apps were (1) targeted at patients with AS; (2) in the Chinese language; (3) available for downloading from the Huawei and/or Apple App Stores; (4) able to run on Android and/or iOS operating systems; (4) incorporated at least one of the following elements of disease management and/or patient education: educational information, medication management, exercise management, psychological strategy, symptom management, etc. Apps were excluded if they were not for patient use or not relevant to AS (eg, apps for other chronic conditions or other use). We also excluded apps that had not been updated since 2018. Android apps were downloaded and tested using the Huawei ATH-AL00 with Android version 5.1.1, and iOS apps were downloaded and tested using the iPhone 11 with iOS 13.3.1 installed.

### App Quality Measures

We systematically searched guidelines and recommendations for mHealth apps and AS management to identify app quality measures [3,4,32-35]. We formulated a list of quality assessment measures from and consistent with guidelines from the EULAR [4,32-34], the American College of Rheumatology [3], and the French Society for Rheumatology [35]. Then, rheumatologists and rheumatology nurses modified the app quality assessment measures. Finally, the list of app quality measures included two domains: basic characteristics and functionalities. Basic characteristics related to the following features extracted: app name, developer, operating system (iOS or Android), version, provider involvement, star rating (out of 5), number of reviews, number of downloads (only available for Android apps), cost, and privacy policy. Functionalities of the included apps were analyzed as follows: basic information record, educational information, communication, symptom tracking, general or psychological health tracking, medication management, visuals or analysis, exercise management, reminder feature, and information sharing. The app quality measures are shown in Table 1.

**Table 1:** App quality measures of apps for patients with ankylosing spondylitis.

Assessment measure			Description and definition
**Basic characteristics**			
	App name	App name as shown in the Huawei and/or Apple app stores	
	Developer	Name of the developer (ie, who developed and uploaded the app)	
	Operating system	iOS and/or Android operating systems	
	Version	Latest update version and date of update	
	Provider involvement	Involvement of relevant health care providers in the design, development, and validation of the app	
	Star rating	Star rating score (out of 5) that users left on the Huawei and/or Apple app stores	
	Number of reviews	Number of reviews that users left on the Huawei and/or Apple app stores	
	Number of downloads	Number of downloads since the app released	
	Cost	Free apps or the cost of apps	
	Privacy policy	Information on how user data are stored and shared	
**Functionalities**			
	Basic information record	Enables users to record their basic information (eg, gender, age, disease history)	
	Educational information	The information content is up to date, scientifically justifiable, acceptable to users, and evidence-based. The content includes disease overview, pathogenesis, treatment goal and options, exercise advice, medication, joint protection, and health advice for daily life	
	Communication	Facilitates patient–health care provider communication and patient-patient communication	
	Symptom tracking	Prompts users to assess their general symptoms as follows: disease activity, pain, fatigue, morning stiffness, and functional ability	
	General or psychological health tracking	Allows users to record information about their general or psychological health, such as sleep quality, depression, anxiety, quality of life, and general health	
	Medication management	Allows users to record medication name, dosing, time, and frequency	
	Visuals or analysis	Displays recorded information as graphs or tables	
	Exercise management	Allows users to record information pertaining to exercise (eg, frequency, time, type)	
	Reminders	Allows users to set reminders for appointments or when to take their medication	
	Information sharing	Allows users to share educational information and/or their disease data with health care providers or others	

### App Rating Using the uMARS

We also used the uMARS to evaluate the quality of apps [36], which is a simplified version of the MARS that has been used to assess the quality of mHealth apps [36,37]. The uMARS is a 20-item measure that comprises 4 objective quality subscales (engagement, functionality, aesthetics, information quality) and 1 subjective quality subscale [36]. We did not include the latter subscale in this study since our aim was to assess the objective quality of apps. All items were rated on a 5-point Likert scale from 1 (inadequate) to 5 (excellent). Mean scores were calculated for each subscale and a mean total score was calculated across all 4 subscales [36]. Apps that scored ≥3 out of 5 on the uMARS were considered to be of acceptable quality, and scores higher than 4 were rated as high quality [38]. Two trained reviewers independently rated the apps using the uMARS. Subsequently, they discussed inconsistencies and doubts about the apps and reached a consensus on the final uMARS scores.

### Data Abstraction

Two reviewers independently downloaded the apps that met the inclusion criteria and evaluated them using our list of quality assessment measures. In addition, they rated the app quality using the uMARS. The main features and quality of all apps were recorded, including basic characteristics, functional features, and uMARS rating.

## Results

### App Selection

**Figure 1:**
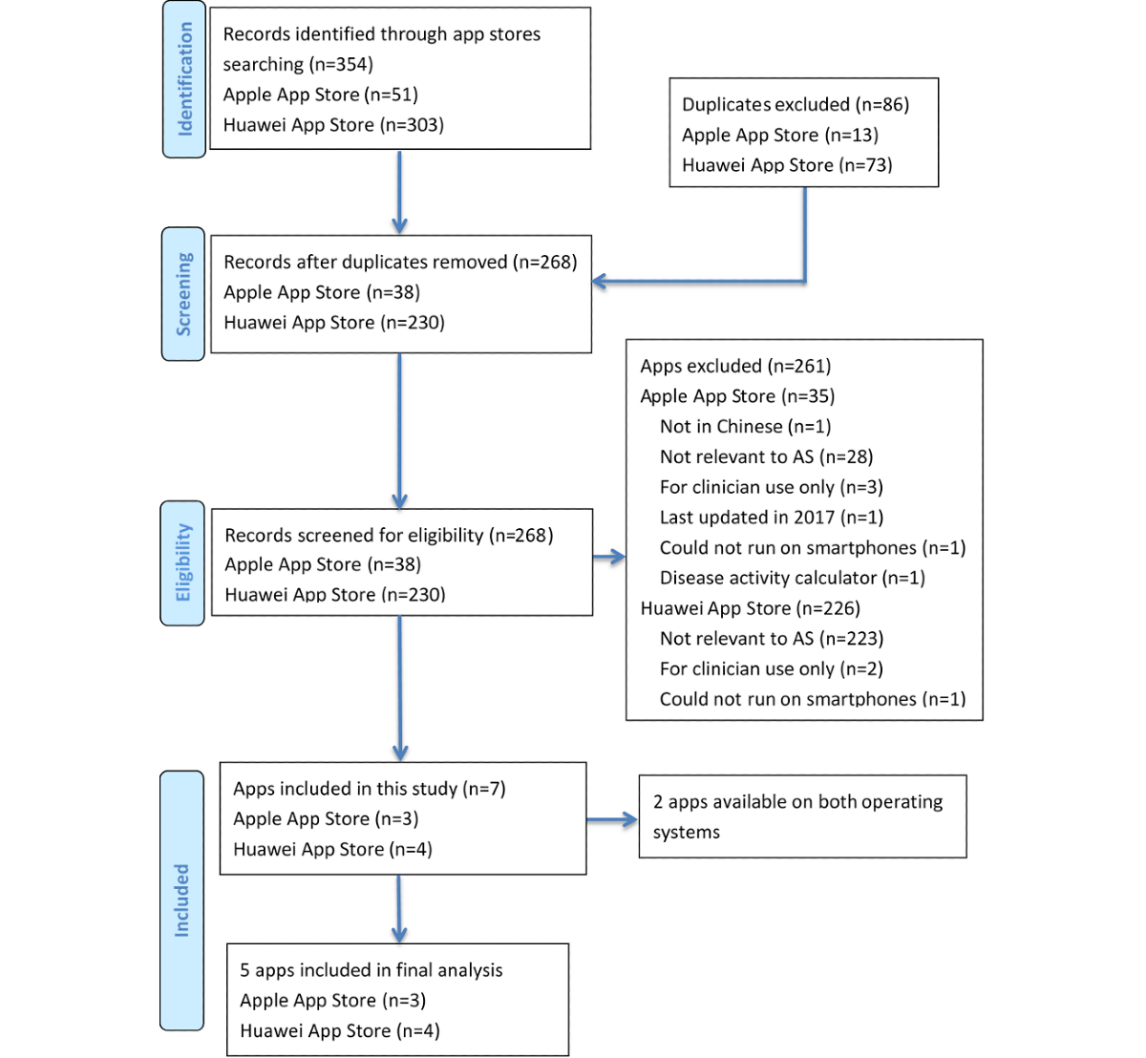
Flow diagram for the systematic search and selection of apps from the Apple and Huawei app stores. AS: ankylosing spondylitis.

Our searches from the Apple App Store retrieved 51 apps. We excluded apps that were duplicates (n=13), not in Chinese (n=1), not relevant to AS (eg, many apps focused on other chronic conditions or provided general health information) (n=28), only for clinician use (n=3), disease activity calculators (n=1), or last updated in 2017 (n=1). Of the remaining 4 apps, 1 app was not able to run on the iOS operating system and was excluded from the study, leaving 3 apps for the final analysis. A total of 303 Android apps were retrieved from the Huawei App Store. Of these, 299 apps were excluded because they were duplicates (n=73), not relevant to AS (eg, many apps focused on other chronic conditions, or were intended for other use including online shopping, work, and games) (n=223), only for clinician use (n=2), or could not run on the Android operating system (n=1). Four Android apps were included for analysis. A total of 5 apps were included in the final analysis since 2 apps were available on both the Android and iOS operating systems (Figure 1). Since the 2 apps had the same functionalities on both the iOS and Android operating systems, they were described once. The 5 apps were iRheuma, Wen Wen Feng Shi, Feng Shi Mian Yi Kou Dai Shu, Lei Feng Shi Hu Zhu, and Jian Feng Yuan. iRheuma is an app by the SSDM, which is part of a series of doctor-patient interactive apps developed for the self-management of patients with chronic diseases.

### App Characteristics

The basic characteristics of the app, based on the app quality measures, are summarized in Tables 2 and 3. All apps were specific to people with AS, but some apps were also relevant to other rheumatic diseases, such as rheumatoid arthritis and gout. Two apps (40%) were developed by companies, and 2 apps (40%) by medical institutions. Four apps (80%) were last updated in 2020, and 1 app was updated in 2018. Only 1 app, Feng Shi Mian Yi Kou Dai Shu, described medical professionals involvement in the development of this app. However, we did not know if patients with AS and/or health care providers were involved in the development of the other apps since these apps did not provide information about their design and development. Two app (40%) received no review, and there was only 1 app (20%) with more than 10 app store reviews. Of the 4 Android apps with available download data, 3 (60%) were downloaded more than 10,000 times, and 1 (20%) was downloaded 450,000 times. All apps were free for users, and most apps (n=4, 80%) had a privacy policy.

**Table 2:** Operating system, developer, version, and health care provider involvement of the included apps.

App name	Operating system	Developer	Date of latest update	iOS version	Android version	Provider involvement
iRheuma	iOS, Android	Shanghai Gete Internet Technology Co, Ltd	Dec 2020	3.12.4	3.12.4	N/A^a^
Wen Wen Feng Shi	iOS, Android	Shanghai Wenyun Biotechnology Co, Ltd	Dec 2020	3.2.4	3.2.3	N/A
Feng Shi Mian Yi Kou Dai Shu	iOS	St. Bear Inc	May 2020	3.1	—^b^	Yes
Lei Feng Shi Hu Zhu	Android	Hu Ze Min Rheumatoid Hospital of TCM	Jul 2020	—	3.8.7	N/A
Jian Feng Yuan	Android	Not stated	Dec 2018	—	2.1	N/A

^a^N/A: not applicable.

^b^Not available.

**Table 3:** Star rating, number of reviews and downloads, cost, and privacy policy of the included apps.

App name	Star rating	Reviews, n	Downloads, n	Cost	Privacy policy
iRheuma	4.2 (iOS); 4.3 (Android)	54 (iOS);10 (Android)	450,000	Free	Yes
Wen Wen Feng Shi	4.4 (iOS); 5 (Android)	28 (iOS); 1 (Android)	90,000	Free	Yes
Feng Shi Mian Yi Kou Dai Shu	N/A^a^	0	N/A	Free	Yes
Lei Feng Shi Hu Zhu	3.7	2	20,000	Free	Yes
Jian Feng Yuan	N/A	0	<10,000	Free	No

^a^N/A: not applicable.

### App Functionalities

The functionalities of the included apps, based on the app quality measures, are shown in Table 4. All apps provided educational information about AS, including disease overview, pathogenesis and cause, symptoms, pharmacological treatment, exercise, and health advice, but none were to tailored individual needs and preferences. Four apps (80%) updated their educational information. Of the 5 included apps, only Wen Wen Feng Shi provided educational materials, including the pathogenesis, cause and treatment of AS, health advice on exercise and lifestyle, and updated medical news.

**Table 4:** Functionalities of the included apps.

Functionality	iRheuma	Wen Wen Feng Shi	Feng Shi Mian Yi Kou Dai Shu	Lei Feng Shi Hu Zhu	Jian Feng Yuan
Educational information	✓^a^	✓	✓	✓	✓
Basic information record	✓		✓	✓	
Communication	✓				✓
Symptom tracking	✓				✓
General or psychological health tracking	✓				
Medication management	✓				
Laboratory result tracking	✓				
Visuals or analysis	✓				
Exercise management					
Reminders	✓				
Information sharing	✓	✓		✓	

^a^Check mark indicates presence of feature.

Three apps (60%) allowed users to record their basic information, such as gender, age, health history, and medical record. Two apps (40%) had a communication feature. iRheuma enabled users to directly consult doctors through text message, telephone call, and video call, but the communication function did not run well because many doctors did not provide online consultation services. Jian Feng Yuan only supported patient-patient communication via a message board.

Symptom tracking was available for 2 apps (40%): iRheuma and Jian Feng Yuan. iRheuma assessed users’ disease activity and functional ability using validated instrument (ie, Ankylosing Spondylitis Disease Activity Score [ASDAS]; Bath Ankylosing Spondylitis Disease Activity Index [BASDAI]; Bath Ankylosing Spondylitis Functional Index [BASFI]). If patients inputted their data, iRheuma could give patients feedback on disease activity and function, and provide individual health advice. Jian Feng Yuan used only the ASDAS to assess users’ disease activity and calculated an ASDAS score.

iRheuma also provided general or psychological health tracking, medication management, laboratory result tracking, visuals or analysis, and reminders. iRheuma used validated instruments (Hospital Anxiety and Depression Scale, Pittsburgh Sleep Quality Index, Medical Outcomes Study Short Form 36-item Health Survey) to assess patients’ health and to give them feedback and tailored health advice. iRheuma enabled users to record their medication and laboratory results, and to set reminders to take their medication. This app could create graphs from user-reported data to track users’ symptoms and laboratory results.

No app had an exercise management feature. Three apps (60%) supported an information-sharing function, but they only allowed users to share health information with other people via WeChat, Tencent QQ, and email. Some functions of the included apps did not run well due to technical issues. Among these apps, iRheuma was downloaded 450,000 times more than other apps.

### Additional Functionalities

Two apps (40%) supported online shopping. Users could purchase medications, medical devices, and medical books on these apps. One app provided questions about the Modified New York Classification Criteria for AS [39] to screen for AS. Two apps (40%) enabled users to make appointments with doctors. Most apps (4/5, 80%) linked to a WeChat public account with content and functions similar that in the app.

### App Rating Based on the uMARS

Table 5 shows the uMARS ratings for all included apps. The overall uMARS scores for the apps ranged from 2.7 to 4.2. Three (60%) apps scored more than 3 out of 5; only iRheuma (4.2) app scored than 4 out of 5. Information quality scores (2.8-4.5) showed the greatest variability. The engagement scores (2.2-3.8) were the lowest of the 4 subscales.

**Table 5:** The user version of the Mobile App Rating Scale (uMARS) scores of the included apps.

App name		uMARS subscale score					Overall score
		Engagement	Functionality	Aesthetics	Information quality		
**iRheuma**							
	iOS	3.8	4.3	4.3	4.5	4.2	
	Android	3.8	4.3	4.3	4.5	4.2	
**Wen Wen Feng Shi**							
	iOS	2.6	3.5	3.7	3.5	3.3	
	Android	2.6	3.5	3.7	3.5	3.3	
**Feng Shi Mian Yi Kou Dai Shu**							
	iOS	2.4	3.3	3.0	2.8	2.9	
**Lei Feng Shi Hu Zhu**							
	Android	2.2	3.0	2.7	2.8	2.7	
**Jian Feng Yuan**							
	Android	2.8	3.0	3.7	3.3	3.2	

## Discussion

### Principal Findings

This study utilized a systematic approach to identify and evaluate 5 mHealth apps for AS management in China. We found only 1 app (with an overall uMARS score of 4.2) that provided comprehensive functions that adhered to evidence-based guidelines for AS management. This result was line with previous reviews of apps on gout and rheumatoid arthritis [20,40]. Most apps in the study did not provide further information regarding the development process of the apps. Similarly, Najm et al [18] also found that the development process of most apps were not sufficient or were not described in the existing literature, which might raise questions about apps’ credibility. Thus, international guidelines and regulations for the design, development, and validation of mHealth apps are needed in the future. Additionally, our results were in line with previous evidence that most apps designed for patient use did not involve health care providers or patients in the development stage [18]. Prior evidence suggested that the development and validation of self-management apps should involve target users and health care providers [32,41].

The EULAR has recommended that mHealth apps should be relevant and tailored to the individual needs of people with rheumatic and musculoskeletal disease [32,34]. In our study, educational information in most of the apps was not tailored to patients’ needs and preferences. It may be that patients and health care professionals were not involved in the development of the apps [18]. Moreover, the educational information in some apps was not divided into different disease modules, which may make it difficult for users to find information on AS. We did not systematically evaluate patient acceptability of these apps due to lack of a quantitative assessment. Future mobile apps should provide evidence-based educational information and increase apps’ usability based on patients’ needs and health care professionals’ perspectives.

Many symptom-tracking apps for rheumatic disease did not use validated instruments [16,29]; only 2 apps in this study used validated instruments to track patients’ symptoms. iRheuma used the ASDAS, BASDAI, and BASFI to track users’ disease activity and functional ability. Moreover, iRheuma was also able to provide feedback on users’ conditions and encourage users to consult with doctors, which may enable users to better understand their conditions and monitor their disease. Another app, Jian Feng Yuan, could calculate users’ disease activity (using the ASDAS). Both apps lacked the ability to directly transmit data to health care providers. Although evidence has suggested that mobile apps that use a validated instrument and have a tracking function may be useful for patients with arthritis for symptom monitoring [16,29], most of these apps, as well as the ones from our study, were not assessed in clinical trials. Thus, future studies should explore the effects of these apps on health and economic outcomes.

In this study, 1 app had a reminder feature. Prior work has suggested that apps with such a function may be effective in improving medication adherence in nonadherent patients [42]. It is important to integrate this function into clinical practice. However, the feasibility and effects of reminder apps have not been well studied. Future studies should explore the efficacy of reminder apps in large populations.

Of the included 5 apps, only iRheuma provided comprehensive functions, including direct communication, health tracking, medication management, and laboratory results tracking. These functions may help patients better manage their conditions. Additionally, we found that iRheuma was the most frequently downloaded app of the 5 apps, indicating that multifunction apps may be attractive for patients with AS. Luo et al [20] revealed that only 25% of reviewed apps provided symptom tracking and education for patients about management strategies [20]. Although international guidelines have revealed the importance of exercise for AS management [3,43,44], none of the apps provided this function. Future studies should develop comprehensive, evidence-based apps for patients with AS.

The overall uMARS scores ranged from 2.7 to 4.2, indicating inconsistent quality among the included apps. This result is similar to previous studies reviewing mHealth apps [29,45]. Only 1 app (iRheuma) provided comprehensive functions for AS management and was considered high quality based on the uMARS. The 2 highest scoring apps, iRheuma (4.2) and Wen Wen Feng Shi (3.3), were most frequently downloaded, indicating that the high-quality apps may be useful for targeted users. Engagement scores were lower compared to the other 3 subscales, and nearly half (2/5, 40%) of the apps were considered to be of poor quality. Thus, future studies and practice should improve apps’ quality for AS management, especially engagement quality.

### Strengths and Limitations

Strengths of this study included the assessment of a broad range of app characteristics, including basic characteristics, functionalities, and app ratings using uMARS scores. This study also had several limitations. One limitation is that we only focused on Chinese-language apps available on the two most popular operating systems (Android and iOS), and thus missed apps available in other languages or on other operating systems. Although WeChat public accounts have been increasingly used as mHealth tools in China [46], we did not include WeChat public accounts for AS management, such as the Smart-phone SpondyloArthritis Management System [11]. We searched for apps in two of the most popular app stores (the Huawei and Apple App Stores) for Android and iOS operating systems in China, but apps exclusively in other app stores (eg, Microsoft) were not included in this study. This study also only focused on publicly available apps. Apps available in the published literature were not included since limited published works have focused on apps for AS management. The uMARS does not focus on AS management apps. Thus, we developed a list of app quality assessment measures based on evidence-based guidelines and recommendations for mHealth apps and AS management. The app quality measures have not been validated, which may limit the findings of this study.

### Conclusions

This study found a lack in high-quality apps available to assist in the management of AS in China. Only 1 out of the 5 apps was of high quality and provided comprehensive functions to help patients manage their conditions. Most apps lacked key features for disease management, such as symptom tracking, medication management, and reminders. This study only assessed the app quality and did not evaluate the usability and efficacy of the included apps. Future studies and clinical practice should explore the efficacy and feasibility of mHealth apps. International guidelines and regulations for the design, development, validation, and implementation of mHealth apps are also needed in the future. In the meantime, health care providers, patients with AS, and app developers should collaborate to develop high-quality, evidence-based apps that consider patients’ needs and health care professionals’ perspectives.

## Abbreviations

AS: ankylosing spondylitis
ASDAS: Ankylosing Spondylitis Disease Activity Score
BASDAI: Bath Ankylosing Spondylitis Disease Activity Index
BASFI: Bath Ankylosing Spondylitis Functional Index
EULAR: European League Against Rheumatism
MARS: Mobile App Rating Scale
mHealth: mobile health
PRISMA: Preferred Reporting Items for Systematic Reviews and Meta-Analyses
SSDM: Smart System of Disease Management
uMARS: Mobile App Rating Scale, user version
